# Comprehensive clinical and genetic architecture of familial amyotrophic lateral sclerosis in China: A 15-year cohort study with 302 families

**DOI:** 10.4103/NRR.NRR-D-24-00701

**Published:** 2025-01-13

**Authors:** Wei Zheng, Lu Xu, Jinling Cai, Jinwen Hou, Lu Chen, Nan Zhang, Siyan Zhan, Dongsheng Fan, Ji He

**Affiliations:** 1 Department of Neurology, Peking University Third Hospital, Beijing, China; 2 Beijing Key Laboratory of Biomarker and Translational Research in Neurodegenerative Diseases, Beijing, China; 3 Key Laboratory for Neuroscience, National Health Commission/Ministry of Education, Peking University, Beijing, China; 4 Research Center of Clinical Epidemiology, Peking University Third Hospital, Beijing, China; 5 Key Laboratory of Epidemiology of Major Diseases (Peking University), Ministry of Education, Beijing, China; 6 School of Basic Medical Sciences, Peking University, Beijing, China; 7 Department of Epidemiology and Biostatistics, School of Public Health, Peking University, Beijing, China; 8 Center for Intelligent Public Health, Institute for Artificial Intelligence, Peking University, Beijing, China; 9 Biomedical Pioneering Innovation Center (BIOPIC), Peking University, Beijing, China; 10 Changping Laboratory, Beijing, China

**Keywords:** China, cohort, epidemiological, familial amyotrophic lateral sclerosis, gene-level burden analysis, genetic, genotype, group-based trajectory model, pathogenic, phenotype

## Abstract

The growing recognition of the role of genetics in the development of amyotrophic lateral sclerosis is evident. However, there has yet to be a comprehensive analysis of the clinical characteristics and genetics of familial amyotrophic lateral sclerosis in an Asian population. This study aimed to provide an in-depth analysis of the clinical features and genetic spectrum of familial amyotrophic lateral sclerosis over 15 years in a clinic-based cohort of patients from the Chinese mainland. Enrollment of 302 amyotrophic lateral sclerosis families from 28 provinces was undertaken from January 2008 to September 2023. A group-based trajectory model for disease progression based on amyotrophic lateral sclerosis Functional Rating Scale-Revised (ALSFRS-R) scores was validated using bootstrap internal validation in patients with familial amyotrophic lateral sclerosis, as well as patients with sporadic amyotrophic lateral sclerosis (matched at a 1:4 ratio, with replacement). DNA samples from 244 index patients were screened for variants in the pathogenic genes *SOD1*, *FUS*, *TDP43*, and *C9ORF72*, of which 146 were also subjected to genome-wide next-generation sequencing. Gene-level burden analysis was used to evaluate the distribution of rare variants in the cohort. We found that rapid dynamic disease progression was associated with an older age at onset, shorter diagnostic delay, lower body mass index, bulbar onset, and ≥ 1 affected first-degree relative. Certain attributes, such as age at onset and time from onset to diagnosis, had comparable impacts on the clinical progression trajectories of both familial amyotrophic lateral sclerosis and sporadic amyotrophic lateral sclerosis. Harboring pathogenic/likely pathogenic variants in amyotrophic lateral sclerosis-causative genes reduced the age of onset of familial amyotrophic lateral sclerosis. Among the patients with familial amyotrophic lateral sclerosis, 17.8% possessed ≥ 2 pathogenic/likely pathogenic variants. Sequencing kernel association test analysis showed that the SOD1 rare variant burden (*P* = 1.3e−15) was associated with a significant risk of familial amyotrophic lateral sclerosis. Our findings conclusively confirmed the clinical features and genetic spectrum of familial amyotrophic lateral sclerosis over 15 years in a clinical cohort from China, contributing to a deeper understanding of genotype–phenotype relationships in familial amyotrophic lateral sclerosis. This comprehensive evaluation of specific clinical characteristics, clinical prognosis, and genetic variants of amyotrophic lateral sclerosis based on detailed clinical and genetic information may lead to the development of genotype-specific treatment approaches.

## Graphical abstract

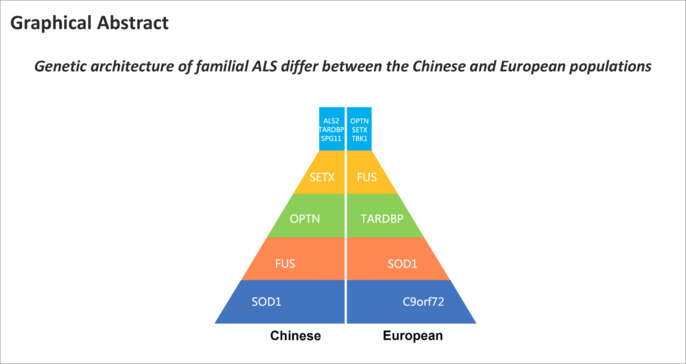

## Introduction

Amyotrophic lateral sclerosis (ALS) is a fatal neurodegenerative disease characterized by progressive muscle weakness that predominantly affects upper and lower motor neurons (Tang and Fan, 2022). Patients usually die of neuromuscular respiratory failure 2–5 years after diagnosis (Goutman et al., 2022). Mutations in genes associated with ALS can also present as frontotemporal dementia (FTD), which is part of the same spectrum as ALS (Dols-Icardo et al., 2018). ALS is usually classified into familial ALS (fALS), which accounts for 5%–20% of ALS cases (depending on the definition of fALS), and sporadic ALS (sALS) (Byrne et al., 2013; Al-Chalabi et al., 2017). Since 1993, at least 40 genes have been implicated in the pathogenesis of ALS (Brenner and Freischmidt, 2022). *C9ORF72*, *SOD1*, *FUS*, and *TARDBP* are the four most commonly mutated genes among European and Asian patient populations (McCann et al., 2017). These known ALS-associated mutations explain two-thirds of fALS cases and approximately 10% of sALS cases (Chia et al., 2018; Brenner and Freischmidt, 2022). Notably, beyond contributing to our understanding of the basic molecular mechanisms of ALS, genetic discoveries may also lead to improvements in genotype-specific therapeutic approaches. These include antisense oligonucleotide-based therapies, such as ION363, which is being tested for FUS-ALS (Korobeynikov et al., 2022), and Tofersen, which is already available for *SOD1*-ALS (Miller et al., 2022). Therefore, a comprehensive overview of the mutational landscape in a large cohort of fALS patients may deepen our understanding of the pathogenesis and treatment of ALS.

The genetic architectures of European and Asian patient populations are distinct (Zou et al., 2017). Research focused on the Chinese ALS population indicates a trend toward earlier disease onset (Wei et al., 2015, 2019). Current knowledge about the genes that cause ALS originates primarily from people of European ancestry (i.e., Europe, the United States, Canada, and Australia) (Pfister et al., 2013; McCann et al., 2017; Ryan et al., 2018). Until recently, information on the clinical features and genetic architecture of fALS in Asian patients, which represent one-fifth of the world’s population, has been limited. However, previous fALS cohort studies were limited in sample size and lacked long-term follow-up (Liu et al., 2019, 2021; Chen et al., 2020, 2021).

Here, we investigated a total of 302 fALS families from 28 provinces of China to characterize the clinical features and genetic architecture of fALS over 15 years. To the authors’ knowledge, this study represents one of the largest fALS cohorts worldwide, with the longest follow-up period (15 years). We also evaluated the distribution of rare variants in the ALS cohort and in-house controls using gene-level burden analyses. This study provides a comprehensive analysis of the clinical features and genetic spectrum of fALS in the Chinese mainland, with the aims of deepening our understanding of genotype–phenotype relationships in ALS, improving the practice of genetic counseling in the clinical setting, and evaluating the prognosis of ALS disease.

## Methods

### Participants and data collection

For this retrospective study, a total of 338 fALS families from the ALS patient database of Peking University Third Hospital (PUTH) were initially evaluated from January 2008 to September 2023. The fALS diagnosis was based on Byrne’s criteria regarding the presence of at least one first-degree, second-degree, or distant relative with ALS or FTD spectrum disorder (Byrne et al., 2011). The exclusion criteria were incomplete medical records and absence of signed informed consent. For all index patients, baseline clinical data and demographic information were collected during the patient’s first visit to PUTH. Patients were diagnosed ALS in accordance with the revised El Escorial diagnostic criteria, then diagnosed fALS based on Byrne’s criteria (Brooks et al., 2000). We classified the fALS patients into definite, probable and possible fALS categories using Byrne’s criteria. This study categorized patients into three groups based on their family history of ALS. The definite group consisted of patients who had at least two first- or second-degree relatives with ALS. The probable group included patients who had one first- or second-degree relative affected by ALS. The possible group comprised patients who had one distant relative affected by ALS. First-degree relatives referred to parents, offspring, and siblings, while second-degree relatives included grandparents and aunts/uncles. The fALS patients were followed by neurologists through in-person visits or telephone calls every 3 months. Demographic data, including sex, age at onset, diagnostic delay, family history, body mass index (BMI), smoking and drinking history, and history of other diseases, were collected for all patients. All patients underwent testing for routine blood parameters, kidney function, liver function, lung function, lipid profile, and electrolytes, as well as cranial magnetic resonance imaging and electromyography. In recent years, the blood level of neurofilament light chain (NfL) was analyzed in some patients (Zhang et al., 2022a). The Edinburgh Cognitive and Behavioral Assessment Screen was used to assess cognition (Poletti et al., 2024). To assess disease severity throughout the 15-year observation period, we used data from patient-reported outcome measures, such as ALS Functional Rating Scale-Revised (ALSFRS-R) scores (Chen et al., 2021), which were systematically recorded and analyzed every 3 months. Diagnostic delay was defined as the time from symptom onset to diagnosis of ALS by experienced neurologists. Survival time was defined as the time from symptom onset to the endpoint event (death or endotracheal intubation). This study was approved by the Institutional Ethics Committee of PUTH (approval No. 223-02) on September 5, 2017, and was conducted in accordance with the *Declaration of Helsinki*. Each participant provided written informed consent. This study was reported according to the STrengthening the Reporting of OBservational studies in Epidemiology (STROBE) statement (von Elm et al., 2007).

### Trajectory analysis

A group-based trajectory model (GBTM) was used to classify fALS patients by changes in ALSFRS-R scores over time. GBTMs can be used to identify clusters of patients (i.e., trajectory groups) who follow a similar longitudinal trend on an outcome of interest (Nagin and Odgers, 2010). The process of determining the number of trajectory groups for the GBTM involved the following components. (1) There were three basic requirements for statistical indices: the smallest possible absolute value of the Bayesian information criterion; a minimum percentage of patients in each group ≥ 5%; and a *P* value for the highest order of each subgroup ≤ 0.05. The values of these indexes are provided in **Additional Table 1**. (2) Clinical judgments were based on clinical experience, reasonability (e.g., the trajectory cannot experience significant rebound), and interpretability. (3) If the lower-order trajectory equation exhibited good fit, the higher-order trajectory equation was not needed. Statistical criteria (smallest possible absolute value of the Bayesian information criterion and ≥ 5% of patients in each group) and clinical judgments were combined to determine the number of trajectory groups and the order of their corresponding polynomials (i.e., linear, quadratic, cubic). Internal validation for the trajectory analysis was provided by the bootstrap procedure, which involved repeated resampling of the original dataset with random replacement 1000 times to obtain 1000 new datasets. To explore the differences and trends among the trajectory groups (an ordinal variable), the baseline characteristics of different trajectory groups were compared by ordered logistic regression. Additionally, to explore differences in prognosis (tracheostomy or death) among the trajectory groups and among patients with different genetic factors, hazard ratios and 95% confidence intervals were calculated from the multivariate Cox proportional hazards model, adjusted for age at onset, sex, fALS grade, BMI, and site of onset. Through propensity score matching, each fALS patient was matched to four sALS patients by sex and age at onset, with replacement. The GBTM analysis was also performed for the matched sALS patients to compare the trajectories of fALS and sALS.

### Genetic analysis

After obtaining signed consent, peripheral blood was collected and DNA was extracted using standard phenol-chloroform procedures. Owing to the limitations in medical history, DNA samples were available from 244 of the 302 index patients. All 244 DNA samples were screened by Sanger sequencing for *SOD1*, *FUS*, and *TDP43*, and by repeat-primed PCR for *C9ORF72*, prior to 2013. For 146 the 244 patients, DNA samples were also screened by genome-wide next-generation sequencing (NGS). According to previous strategies, assessment of the 42 known ALS-causative genes was undertaken using NGS data from the 146 patients and whole-exome sequencing (WES) data from 1812 in-house controls (**Additional Table 2**; Goutman et al., 2022; Zhang et al., 2022b). All nonsynonymous variants were assigned and filtered using data from the 1000 Genomes Project catalog and the Exome Aggregation Consortium (ExAC) database. The variants of the 42 ALS-causative genes were classified as pathogenic (P), likely pathogenic (LP), or variant of uncertain significance (VUS), in accordance with the American College of Medical Genetics and Genomics recommendations (Richards et al., 2015; Chen et al., 2021).

**Additional Table 2:** Basic characteristics of 42 ALS-causative genes investigated in this study

Gene	Chromosomal locus	Mode of inheritance	Canonical transcript	Transcript length (kbp)	Putative pathway/function	Strength in ALSod
*ALS2*	2q33.1	AR	NM_020919.3	6.64	Vesicle trafficking	Tenuous
*ANG*	14q11.2	AD	NM_001145.4	1.22	Angiogenesis	Moderate
*ANXA11*	10q22.3	AD	NM_145869.1	6.97	Cell membrane repair, vesicle trafficking	Definitive
*C21orf2*	21q22.3	AD	NM_004928.2	2.23	Cilia formation, actin structure	Unassigned
*C9orf72*	9p21.2	AD	NM_018325.3	3.20	Autophagy, proteostasis	Definitive
*CCNF*	16p13.3	AD	NM_001761.2	4.29	Proteostasis, coordination of the cell cycle	Strong
*CHCHD10*	22q11.23	AD	NM_213720.3	1.17	Autophagy, neuroinflammation	Definitive
*CHMP2B*	3p11.2	AD	NM_014043.3	2.60	Proteostasis, vesicular trafficking	Moderate
*CTSF*	11q13.2	AD/AR	NM_003793.3	2.04	Lysosomal pathway	Unassigned
*DCTN1*	2p13.1	AD	NM_004082.4	4.50	Proteostasis, retrograde axonal transport	Tenuous
*DNAJC7*	17q21.2	AD	NM_003315.3	2.02	Proteostasis	Moderate
*ELP3*	8p21.1	RF	NM_018091.5	3.45	Ribostasis, cytoskeletal integrity	Tenuous
*ERBB4*	2q34	AD	NM_005235.2	12.1	Mitogenesis and differentiation	Moderate
*FIG4*	6q21	AD	NM_014845.5	3.01	Vesicular trafficking	Moderate
*FUS*	16p11.2	AD	NM_004960.3	2.05	Ribostasis	Definitive
*GLT8D1*	3p21.1	AD	NM_018446.3	2.10	Proteostasis, glycosyltransferase	Tenuous
*HNRNPA1*	12q13.13	AD	NM_031157.2	1.84	RNA-binding protein	Definitive
*HNRNPA2B1*	7p15.2	AD	NM_031243.2	3.66	RNA-binding protein	Tenuous
*KANK1*	9p24.3	NA	NM_001256876.1	5.58	Cytoskeleton formation	Unassigned
*KIF5A*	12q13.3	AD	NM_004984.2	5.82	Kinesin microtubule motor protein	Definitive
*LGALSL*	2p14	NA	NM_014181.2	3.93	Galectin-related protein	Unassigned
*MATR3*	5q31.2	AD	NM_018834.5	4.84	RNA-binding protein	Tenuous
*MFSD8*	4q28.2	AD	NM_152778.2	4.51	Lysosomal pathway	Unassigned
*NEFH*	22q12.2	AD	NM_021076.3	3.78	Axonal transport	Tenuous
*NEK1*	4q33	AD	NM_012224.2	5.65	DNA damage repair	Definitive
*OPTN*	10p13	AD/AR	NM_001008213.1	3.52	Autophagy, vesicle trafficking, NF-kB signal transduction	Definitive
*PFN1*	17p13.2	AD	NM_005022.3	1.29	Axonal growth and transport, cytoskeletal organization	Definitive
*PRPH*	12q13.12	AD	NM_006262.3	3.24	Cytoskeletal organization	Tenuous
*SETX*	9q34.13	AD	NM_015046.5	11.1	Ribostasis	Tenuous
*SIGMAR1*	9q34.13	AR	NM_005866.2	1.66	Signal transduction amplification	Tenuous
*SOD1*	21q22.11	AR/AD	NM_000454.4	0.97	Superoxide anion detoxifying enzyme	Definitive
*SPG11*	15q14	AR	NM_025137.3	7.77	Vesicular transport, autophagy	Tenuous
*SPTLC1*	9q22.31	AD	NM_006415.3	1.89	Lipid metabolism	Unassigned
*SQSTM1*	5q35.3	AD	NM_003900.4	2.99	Autophagy, NF-kB signal transduction	Moderate
*TARDBP*	1p36.22	AD	NM_007375.3	2.75	RNA-binding protein	Definitive
*TBK1*	12q14.2	AD	NM_013254.3	3.26	Autophagy, inflammation	Definitive
*TBP*	6q27	AD	NM_003194.4	1.90	RNA processing	Unassigned
*TIA1*	2p13.3	AD	NM_022173.2	4.63	Ribostasis	Tenuous
*TUBA4A*	2q35	AD	NM_006000.2	2.16	Axonal transport, cytoskeletal organization	Strong
*UBQLN2*	Xp11.21	XD	NM_013444.3	4.23	Proteostasis	Definitive
*VAPB*	20q13.32	AD	NM_004738.4	7.94	Proteostasis	Definitive
*VCP*	9p13.3	AD	NM 007126.3	4.38	Proteostasis	Definitive

AD: Autosomal dominant; ALS: amyotrophic lateral sclerosis; AR: autosomal recessive; XD: X-linked dominant.

### Burden analysis

The collective risk of rare variants for ALS (excluding *C9ORF72*) was investigated using the sequencing kernel association test (SKAT), implemented in the R package AssotesteR. Rare variants identified from WES of the 1812 in-house controls (routinely used anonymous healthy control samples) were used as the control group. According to previous strategies, rare damaging variants with a minor allele frequency (MAF) of < 0.01% in our dataset were analyzed in each gene from 139 patients and 1812 in-house controls, after quality control, using data from ExAC and DiscovEHR (for deleterious insertions) (Farhan et al., 2019; Li et al., 2020). A *P* value < 5e–8 was considered to indicate variants with statistical significance.

### Statistical analysis

Statistical analysis included the Student’s *t*-test to compare normally distributed continuous variables, the Kruskal‒Wallis test to compare non-normal continuous variables, and the chi-square test to compare categorical variables between the two groups. To visually evaluate survival differences within subgroups, Kaplan‒Meier curves were employed, with log-rank testing of differences. A *P* value < 0.05 with a two-tailed test was considered to indicate statistical significance. Statistical analysis was conducted using GraphPad Prism 8.4.0 (GraphPad Software, San Diego, CA, USA; www.graphpad.com) and Stata 16.0 (StataCorp LLC, College Station, TX, USA; www.stata.com) software.

## Results

### Demographics and clinical features

While 338 fALS families met the inclusion criteria for this study, 36 were excluded because of incomplete medical records or absence of signed informed consent (**Additional Figure 1**). Ultimately, data on 302 fALS index patients from 28/32 provinces were recorded in the PUTH ALS patient database between January 2008 and September 2023 (**Additional Figure 1** and **Additional Table 3**). Of the 302 index patients in the cohort, 130 (43.0%) had upper limb-onset ALS, 128 (42.4%) had lower limb-onset ALS, and 44 (14.6%) had medulla oblongata-onset ALS (**Additional Table 4**). The mean age at onset was 46.9 (range, 45.5–48.4) years. The male:female ratio was 1.5. We identified FTD in 11.6% (35/302) of the fALS patients based on medical records collected for this study. Of these 35 patients, behavioral variant FTD was the most prevalent subtype, accounting for 60% of FTD cases. Throughout the study period, all subjects underwent comprehensive clinical and neurophysiological evaluations to ensure accurate diagnosis. Notably, no patients in our cohort exhibited the typical hallmark features of Hirayama disease, such as the insidious onset of muscle weakness in the distal upper limbs, particularly in adolescent males, nor did we observe the characteristic findings on cervical spine imaging during neck flexion in any patients. The demographic and clinical characteristics of the study participants are shown in **Additional Table 4**.

**Additional Table 3:** Geographical distribution of fALS patients in China

Province/City/Autonomous Region	*n*	Percentage
Beijing	50	16.56%
Hebei	32	10.60%
Henan	25	8.28%
Inner Mongolia	21	6.95%
Shandong	21	6.95%
Liaoning	18	5.96%
Jiangsu	15	4.97%
Heilongjiang	13	4.30%
Anhui	10	3.31%
Jiangxi	10	3.31%
Hunan	10	3.31%
Hubei	9	2.98%
Guangdong	9	2.98%
Shanxi	9	2.98%
Jilin	8	2.65%
Fujian	7	2.32%
Zhejiang	7	2.32%
Tianjin	6	1.99%
Guizhou	4	1.32%
Shanghai	3	0.99%
Gansu	3	0.99%
Guangxi	2	0.66%
Shanxi	2	0.66%
Hainan	2	0.66%
Xinjiang	2	0.66%
Sichuan	2	0.66%
Ningxia	1	0.33%
Chongqing	1	0.33%

fALS: Familial amyotrophic lateral sclerosis.

**Additional Table 4:** Demographic and clinical characteristics of fALS patients

	Onset location			
	Upper limb	Lower limb	Medulla oblongata	Total
Total *n* (%) from 302 families	130 (43.0)	128 (42.4)	44(14.6)	302(100)
Male	86 (47.3)	68 (37.4)	28(15.4)	182
Female	44 (36.7)	60 (50.0)	16(13.3)	120
M:F ratio	2.0:1	1.1:1	1.8:1	1.5:1
Age at onset (yr)				
Mean (95% CI)	47.7(45.4-49.9)	45.4 (43.1-47.6)	49.4 (45.5-53.4)	46.9(45.5-48.4)
Median (range)	47.5 (14-80)	45.0(13-74)	51.5 (21-73)	47.0(13-80)
Diagnostic delay from symptom onset (mon)				
Mean (95% CI)	26.6(20.0-33.1)	38.8(30.6-47.1)	20.7(12.8- 28.5)	30.9(26.3-35.6)
Median (range)	14.5(2-313)	20.0 (1-240)	12.5 (2-125)	16.0(1-315)
BMI (kg/m^2^)				
Mean (95% CI)	23.2(22.6-23.8)	23.0 (22.3-23.6)	21.8(20.9-22.8)	22.9(22.5-23.3)
Median (range)	22.9(13.9-31.1)	22.8(14.6-31.2)	21.7(13.8-28.7)	22.7(13.8-31.2)

BMI: Body mass index; CI: confidence interval; fALS: familial amyotrophic lateral sclerosis;

### Clinical trajectories of the familial amyotrophic lateral sclerosis cohort

**Figure 1:**
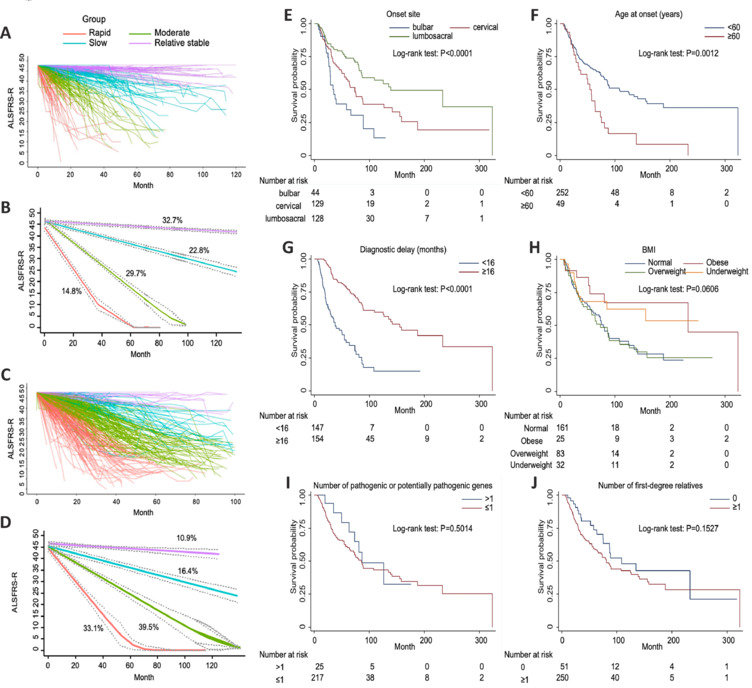
Trajectories of ALSFRS-R scores in the four trajectory groups identified by the GBTM and Kaplan‒Meier survival curves by variable. (A) Changing patterns of ALSFRS-R scores in each fALS patient in the following four trajectory groups identified by GBTM: rapid group (red), moderate group (green), slow group (blue), and relatively stable group (purple). (B) Proportion of fALS patients in each fALS trajectory group. (C) Changing patterns of ALSFRS-R scores in each sALS patient in the four trajectory groups identified by GBTM: rapid group (red), moderate group (green), slow group (blue), and relatively stable group (purple). (D) Proportion of sALS patients in each sALS trajectory group. (E) Kaplan‒Meier survival curves stratified by onset site: bulbar vs. cervical vs. lumbosacral (*P* < 0.0001). (F) Kaplan‒Meier survival curves stratified by age at onset: < 60 years *vs*. ≥ 60 years (*P* = 0.0012). (G) Kaplan‒Meier survival curves stratified by diagnostic delay: < 16 months *vs*. ≥ 16 months (*P* < 0.0001). (H) Kaplan‒Meier survival curves stratified by BMI: underweight (< 18.5) *vs*. normal (≥ 18.5 to < 24) *vs*. overweight (≥ 24 to < 28) *vs*. obese (≥ 28) (*P* < 0.0001). (I) Kaplan‒Meier survival curves stratified by the number of pathogenic or potentially pathogenic genes: > 1 *vs*. ≤ 1 (*P* = 0.5014). (J) Kaplan‒Meier survival curves stratified by the number of first-degree relatives: 0 *vs*. ≥ 1 (*P* = 0.1527). All Kaplan–Meier data analyzed by log-ranked test. ALS: Amyotrophic lateral sclerosis; ALSFRS-R: revised ALS functional rating scale; BMI: body mass index; F: female; fALS: familial amyotrophic lateral sclerosis; GBTM: group-based trajectory model; M: male; sALS: sporadic amyotrophic lateral sclerosis.

All 302 fALS index patients were eligible for GBTM analysis of ALSFRS-R scores over time. Four trajectory groups of disease progression (rapid, moderate, slow, and relatively stable) were identified (**Table 1** and **Figure 1A** and **B**). The trajectories obtained from the bootstrap internal validation procedure were consistent with the main results (**Additional Table 5** and **Additional Figure 2**). Comparison of the baseline characteristics of the four groups revealed that older age at onset (*P* = 0.002), shorter diagnostic delay (*P* < 0.001), lower BMI (*P* = 0.026), at least one affected first-degree relative (*P* = 0.001), and use of riluzole (*P* = 0.018; **Table 1**) were significant factors in the group with rapid disease progression. However, the four groups did not differ in medical history (including hypertension, coronary heart disease, stroke, diabetes, cervical disease, and tumor) or risk factors (including exposure to harmful gas or pesticides, smoking, and drinking). We found a positive correlation between blood neurofilament light chain levels and the rate of ALS progression; however, one-way analysis of variance testing did not show any significant between-group differences. Unexpectedly, there was no significant difference in progression of fALS between patients living in urban and rural settings, possibly due to the relatively small size of our cohort (**Additional Table 6**). Interestingly, we found no statistically significant associations between prognosis and the number of pathogenic/likely pathogenic (P/LP) genes (**Table 1**). These findings have revealed for the first time the occurrence and development of ALS in familial clusters based on a GBTM of progression of ALSFRS-R scores.

**Table 1:** Baseline characteristics of familial ALS patients in the different ALSFRS trajectory groups

Characteristic	Rapid group	Moderate group	Slow group	Relatively stable group	OR (95% CI)	*P*-value
**Sex, male, *n* (%)**	27 (62.79)	72 (63.16)	28 (62.22)	55 (55.00)	0.78 (0.51–1.19)	0.246
**Age at onset, yr, mean (SD)**	49.70 (13.04)	48.09 (12.71)	50.29 (11.74)	42.96 (12.67)	0.97 (0.96–0.99)	0.002
**Diagnostic delay, months, median (IQR)**	8 (6, 12)	11 (6, 19)	40 (24, 52)	30.5 (13, 71.5)	1.04 (1.03–1.05)	< 0.001
**Phenotype, *n* (%)**						
Spinal-onset ALS	20 (86.96)	38 (76.00)	11 (73.33)	25 (96.15)	Ref	
Bulbar-onset ALS	3 (13.04)	10 (20.00)	2 (13.33)	0	0.50 (0.19–1.30)	0.158
FAS	0	1 (2.00)	1 (6.67)	0	1.28 (0.14– 11.55)	0.823
PMA	0	1 (2.00)	1 (6.67)	0	1.28 (0.14–11.55)	0.823
PLS	0	0	0	1 (3.85)	–	–
**fALS classification**						
Possible	0	2 (1.75)	1 (2.22)	8 (8.00)	Ref	
Probable	11 (25.58)	48 (42.11)	12 (26.67)	46 (46.00)	0.23 (0.06–0.87)	0.030
Definite	32 (74.42)	64 (56.14)	32 (71.11)	46 (46.00)	0.14 (0.04–0.55)	0.005
**BMI, kg/m^2^, mean (SD)**	22.84 (2.80)	22.38 (2.85)	22.50 (3.89)	23.69 (3.75)	1.07 (1.01–1.14)	0.026
**Onset site, *n* (%)**						
Cervical	23 (53.49)	47 (41.23)	14 (31.11)	46 (46.00)	Ref	
Lumbosacral	15 (34.88)	40 (35.09)	25 (55.56)	48 (48.00)	1.39 (0.89–2.19)	0.151
Bulbar	5 (11.63)	27 (23.68)	6 (13.33)	6 (6.00)	0.61 (0.33–1.11)	0.105
**Number of P/LP mutated genes**					0.86 (0.62–1.20)	0.370
0	15 (50.00)	55 (56.12)	22 (62.86)	51 (62.96)		
1	12 (40.00)	32 (32.65)	10 (28.57)	21 (25.93)		
2	3 (10.00)	10 (10.20)	3 (8.57)	8 (9.88)		
3	0	1 (1.02)	0	1 (1.23)		
**Number of first-degree relatives**						
0	4 (9.30)	12 (10.53)	9 (20.00)	26 (26.00)	Ref	0.001
≥ 1	39 (90.70)	102 (89.47)	36 (80.00)	74 (74.00)	0.39 (0.22–0.69)	
**NfL, median (IQR)**	78.12 (35.62, 120.64)	76.19 (35.60, 140.52)	42.24 (14.86, 130.12)	32.44 (26.22, 41.81)	1.00 (1.00–1.00)	0.568
**Hypertension, *n* (%)**	8 (21.05)	18 (18.37)	6 (14.63)	16 (20.00)	0.97 (0.55–1.74)	0.929
**Coronary heart disease, *n* (%)**	1 (2.63)	6 (6.12)	1 (2.44)	2 (2.53)	0.69 (0.23–2.08)	0.510
**Stroke, *n* (%)**	0	3 (3.06)	2 (4.88)	1 (1.27)	1.08 (0.29–4.06)	0.906
**Diabetes, *n* (%)**	3 (7.89)	7 (7.22)	5 (12.20)	3 (3.75)	0.74 (0.32–1.70)	0.473
**Riluzole, *n* (%)**	20 (64.52)	39 (54.17)	12 (50.00)	20 (38.46)	0.52 (0.30–0.89)	0.018
**Cervical disease, *n* (%)**	1 (2.63)	2 (2.02)	2 (4.88)	0	0.52 (0.12–2.31)	0.388
**Tumor, *n* (%)**	1 (2.63)	0	0	0	0.81 (0.56–1.18)	0.270
**Exposure to harmful gas, *n* (%)**	6 (15.79)	9 (9.28)	9 (21.43)	7 (8.05)	0.80 (0.41–1.56)	0.517
**Exposure to pesticide, *n* (%)**	2 (5.26)	11 (11.34)	7 (16.67)	7 (8.14)	1.07 (0.54–2.13)	0.850
**Smoking, *n* (%)**	15 (37.50)	33 (31.73)	14 (33.33)	25 (28.41)	0.81 (0.51–1.28)	0.367
**Drinking, *n* (%)**	10 (24.39)	14 (13.46)	7 (16.28)	14 (15.73)	0.82 (0.45–1.48)	0.504

ALS: Amyotrophic lateral sclerosis; ALSFRS-R: revised ALS functional rating scale; BMI: body mass index; CI: confidence interval; FAS: flail arm syndrome; IQR: interquartile range; NfL: neurofilament light chain; OR: odds ratio; P/LP: pathogenic/likely pathogenic; PLS: primary lateral sclerosis; PMA: progressive muscular atrophy; Ref: reference; SD: standard deviation.

**Additional Table 5:** Results of the trajectory analysis of the bootstrap samples *versus* the original results

Trajectory group	Original results		Bootstrap results	

	Linear^a^	Intercept^b^	Linear^a^	Intercept^b^
Rapid group	-0.89953	43.58215	-1.70466	45.9955
Moderate group	-0.49246	46.88173	-0.5468	47.88677
Slow group	-0.16347	46.14331	-0.13776	47.56933
Relatively stable group	-0.03482	46.20899	-0.03736	47.43566

^a^ represents the slope of the trajectory equation. ^b^represents the intercept of the trajectory equation.

**Additional Table 6:** Impact of urban and rural areas on the onset of fALS

Characteristic	Urban group	Rural group	*P*-value
**No. of patients**	206	96	
**Age at onset, yr, mean (SD)**	45.84(11.84)	48.44(12.95)	0.07

fALS: Familial amyotrophic lateral sclerosis; SD: standard deviation.

### Comparison of the clinical trajectories of familial amyotrophic lateral sclerosis and sporadic amyotrophic lateral sclerosis

GBTM analysis was performed to compare the trajectories of all 302 fALS patients and a group of 1:4 matched sALS patients, with the aim to determine the disease occurrence and development of fALS and sALS. Four trajectory groups of disease progression (rapid, moderate, slow, and relatively stable) were identified (**Table 1** and **Figure 1C** and **D**). The coefficients and baseline characteristics of the counterpart trajectory groups were compared using Student’s *t*-test (**Additional Table 7**) and logistic regression analysis (**Table 1** and **Additional Table 8**), respectively. The four trajectories of disease progression—rapid, moderate, slow, and relatively stable—were observed in both the fALS and sALS cohorts (**Table 1** and **Additional Table 8**). For each of the four groups, we conducted a comparison of baseline features between fALS and sALS patients. Certain attributes, including age at onset and time from onset to diagnosis, exhibited significant differences in the clinical progression trajectories of both fALS and sALS patients. Older age at onset (*P* < 0.001) and shorter diagnostic delay (*P* < 0.001; **Additional Table 8**) were significant in the sALS group with faster disease progression, which was consistent with the results in the fALS group. There were no significant between-group differences in medical history (e.g., hypertension, coronary heart disease, stroke, diabetes, and cervical disease) or risk factors (e.g. exposure to harmful gas or pesticides, smoking, and drinking), which was also consistent with the results in fALS patients (**Table 1**). However, while tumors negatively impacted the progression of sALS, they had no statistically significant effects on the clinical progression trajectories of fALS patients (**Table 1** and **Additional Table 8**).

**Additional Table 7:** Comparison of the trajectory groups of patients with fALS and sALS

Trajectory group	fALS		sALS^a^		*P*-value

	Linear^b^	Intercep^c^	Linear^b^	Intercept^c^	
Rapid group	-0.89953	43.58215	-0.87836	37.94611	0.736
Moderate group	-0.49246	46.88173	-0.46135	37.56862	0.329
Slow group	-0.16347	46.14331	-0.21156	35.37118	0.058
Relatively stable group	-0.03482	46.20899	-0.06138	37.13544	0.058

fALS: Familial amyotrophic lateral sclerosis; sALS: sporadic amyotrophic lateral sclerosis. ^a^ One-to-four matching by sex and age at onset with replacement,^b^ represents the slope of the trajectory equation,^c^ represents the intercept of the trajectory equation.

**Additional Table 8:** Baseline characteristics of sALS patients in the different ALSFRS trajectory groups

Characteristic	Rapid group	Moderate group	Slow group	Relatively stable group	OR (95% CI)	P-value
Sex, male, n (%)	68(51.13)	107 (58.15)	17(38.64)	18(66.67)	1.07 (0.74-1.56)	0.722
Age at onset, years, mean (SD)	50.83(14.22)	48.47(15.54)	40.48(13.82)	38.81 (13.45)	0.97 (0.96-0.98)	<0.001
Diagnostic delay, months, median	11 (8, 15)	16 (9, 25)	28(19.5, 47.5)	54(31, 86)	1.05 (1.04-1.06)	<0.001
(IQR)						
Phenotype, n (%)						
Spinal-onset ALS	103 (77.44)	127 (69.02)	35 (79.55)	23 (85.19)	Ref	
Bulbar-onset ALS	21 (15.79)	38 (20.65)	3 (6.82)	3 (11.11)	0.87 (0.53-1.43)	0.256
FAS	5(3.76)	16 (8.70)	5 (11.36)	0 (0)	1.44 (0.70-2.93)	0.132
PMA	3 (2.26)	3 (1.63)	1 (2.27)	1 (3.70)	1.09 (0.28-4.33)	0.857
BMI, kg/m^2^, mean (SD)	22.85 (3.51)	23.16(3.32)	22.41 (3.93)	23.33(2.91)	1.00 (0.95-1.06)	0.866
Onset site, n (%)						
Cervical	72 (55.38)	110(61.11)	32 (72.73)	13 (48.15)	Ref	
Lumbosacral	38 (29.23)	32(17.78)	8(18.18)	11 (40.74)	0.78 (0.48-1.25)	0.296
Bulbar	20 (15.38)	38(21.11)	4 (9.09)	3 (11.11)	0.85 (0.51-1.41)	0.531
Hypertension, n (%)	22 (17.46)	24(13.26)	6(13.64)	3 (11.11)	0.74 (0.43-1.27)	0.278
Coronary heart disease, n (%)	3 (2.38)	4(2.21)	1 (2.27)	0 (0)	0.75 (0.21-2.71)	0.658
Stroke, n (%)	0(0)	1 (0.55)	0 (0)	0 (0)	1.46 (0.06-35.66)	0.815
Diabetes, n (%)	8 (6.35)	10(5.52)	0 (0)	0 (0)	0.48 (0.20-1.13)	0.094
Riluzole, n (%)	60 (54.05)	96 (58.54)	24 (57.14)	12 (48.00)	1.02 (0.68-1.53)	0.920
Cervical disease, n (%)	3 (2.31)	4(2.21)	2 (4.65)	0(0)	1.07 (0.31-3.65)	0.916
Tumor, n (%)	9 (6.92)	27 (14.92)	7(15.91)	6 (22.22)	2.07(1.19-3.62)	0.010
Exposure to harmful gas, n (%)	15 (11.54)	17 (9.44)	5 (11.36)	3 (11.11)	0.93 (0.50-1.73)	0.812
Exposure to pesticide, n (%)	13 (10.08)	18(10.06)	5 (11.36)	1 (3.70)	0.89 (0.47-1.66)	0.705
Smoking, n (%)	38 (37.62)	62 (41.61)	11 (30.56)	10 (45.45)	1.03 (0.67-1.58)	0.905
Drinking, n (%)	18(15.52)	41 (25.15)	6(15.00)	6 (24.00)	1.28 (0.79-2.08)	0.310

ALSFRS-R: revised ALS functional rating scale; ALS, amyotrophic lateral sclerosis; BMI, body mass index; FAS, flail arm syndrome; PLS, primary lateral sclerosis; PMA, progressive muscular atrophy; P/LP: pathogenic/likely pathogenic, IQR: interquartile range; 95% CI, 95% confidence interval; OR, odds ratio; SD, standard deviation.

### Prognostic factors of survival

Next, we analyzed associations between outcome events (respiratory insufficiency or death) and the trajectory groups of fALS patients using a multivariable Cox proportional hazards model. The rapid progression group had a worse prognosis than the other groups (**Additional Table 9**), confirming the trajectory results. In addition, we analyzed the following factors to ascertain potential associations with disease prognosis using Kaplan‒Meier survival analysis (**Figure 1E–J**): onset location, age at onset, sex, diagnostic delay, BMI, number of P/LP genes, and number of first-degree relatives. The Kaplan‒Meier curves shown in **Figure 1E–G** revealed that medulla oblongata onset, older age of onset (≥ 60 years), and a shorter diagnostic delay (< 16 months) were significantly associated with a worse prognosis in fALS patients. Interestingly, there were clear trends of negative correlations between a larger number of P/LP genes or a larger number of first-degree relatives and survival in fALS patients. However, none of these differences reached statistical significance (**Figure 1I** and **J**). Additionally, univariable and multivariable Cox regression models showed that none of the ALS-causative gene mutations influenced the survival of fALS patients (**Additional Table 10**).

**Additional Table 9:** Associations between respiratory insufficiency or death and the trajectory groups according to the multivariable Cox proportional hazards model

Trajectory group	HR (95% CI)
Relatively stable group	Ref
Slow group	2.12(1.05-4.30)
Moderate group	12.89 (6.68-24.87)
Rapid group	27.78(12.89-59.88)

CI: Confidence interval; HR: hazard ratio.

**Additional Table 10:** Survival of fALS patients with different gene mutations

Gene mutations	HR (95% CI)	
	Univariable Cox regression model	Multivariable Cox regression model
*SOD1*	1.18(0.70-1.98)	Reference
*FUS*	2.18(0.87-5.47)	1.23 (0.35-4.35)
*OPTN*	0.53 (0.13-2.19)	0.46 (0.10-2.19)
*SPG11*	2.39 (0.73-7.78)	3.86 (0.73-20.56)
*SETX*	1.35 (0.54-3.37)	0.73 (0.18-2.95)
*TARDBP*	1.74 (0.62-4.89)	2.38 (0.61-9.37)

CI: confidence interval; fALS: familial amyotrophic lateral sclerosis; HR: hazard ratio.

### Genetic architecture of the familial amyotrophic lateral sclerosis cohort

A total of 244 patients were screened for mutations in *SOD1*, *FUS*, *TDP43* and *C9ORF72*, which were detected in 24%, 5.3%, 2.0%, and 0% of our cohort, respectively (**Additional Table 11**). The clinical characteristics, distribution of fALS, and the number of P/LP variants in the 42 ALS-causative genes among 146 index patients are shown in **Table 2**. We found P/LP variants in ALS-causative genes in 60% (88 patients) of the 146 patients. Among the 58 index patients in whom no P/LP gene variants were detected, 14% (21 patients) were genetically unexplained, while 25% (37 patients) carried VUSs (**Table 2**). None of the 1812 in-house controls had any pathogenic variants in the 42 ALS-causative genes. Harboring P/LP variants in ALS-causative genes reduced the age of onset by 5 years in fALS patients (*P* = 0.01; **Table 2**). A greater proportion of fALS patients with P/LP variants in ALS-causative genes had definite fALS, according to Byrne’s criteria (*P* = 0.03; **Table 2**). However, harboring mutated ALS-causative genes did not influence diagnostic delay, BMI, or site of onset.

**Additional Table 11:** Genotype-phenotype correlations in 244 index patients with P/LP variants in major ALS-causative genes

Gene	No. of patients	Sex ratio	Age at onset, mean years (95% CI)	% spinal onset	% bulbar onset	Diagnostic delay from symptom onset, mean months (95% CI)	BMI, mean (95% CI)
*SODI*	59	1.5	44.1 (41.0-46.4)	96.6	3.4	28.9(18.1-39.6)	22.8(21.9-23.7)
*FUS*	13	1.2	40.9(33.7-48.1)	92.3	7.7	17.5 (0.80-34.1)	22.2 (20.1-24.3)
*TARDBP*	5	4.0	53.4(45.9-60.9)	80	20	18.4 (-2.13-38.9)	20.2 (17.9-22.5)
VUS	50	1.3	44.4(41.0-47.9)	86	14	18.5 (13.9-23.1)	23.4 (22.6-24.1)
Unknown	119	1.8	49.4(46.9-51.8)	77.3	22.7	41.8(32.4-51.3)	22.9 (22.3-23.6)

ALS: Amyotrophic lateral sclerosis; BMI: body mass index; CI: confidence interval; P/LP: pathogenic/likely pathogenic; VUS: variant of uncertain significance.

**Table 2:** Clinical characteristics and distribution of familial ALS patients according to the number of P/LP variants in ALS-causative genes

	No. of P/LP variants		*P*-value
	0	≥ 1	
**No. of patients**	58	88	
**Sex ratio (male/female)**	1.8	1.6	
**Age of onset**			0.01
**Mean (95% CI)**	51.0 (47.4–54.6)	46.0 (43.7–48.3)	
**Median (range)**	53.0 (22–80)	45.5 (23–76)	
**Diagnostic delay from symptom onset (mon)**			0.09
Mean (95% CI)	35.3 (22.1–48.6)	23.5 (16.1–30.9)	
Median (range)	19.0 (3–315)	11.5 (1–232)	
**BMI**			0.23
Mean (95% CI)	23.4 (22.5–24.3)	22.8 (22.1–23.4)	
Median (range)	23.1 (16.8–30.5)	22.6 (13.9–29.7)	
**Site of onset**			0.25
Spinal onset, *n* (%)	44 (75.9)	81 (92.0)	
Bulbar onset, *n* (%)	14 (24.1)	7 (8.0)	
**fALS classification**			0.03
Definite, *n* (%)	32 (55.2)	49 (55.7)	
Probable, *n* (%)	25 (43.1)	35 (39.8)	
Possible, *n* (%)	1 (1.7)	4 (4.5)	

ALS: Amyotrophic lateral sclerosis; CI: confidence interval; fALS: familial ALS; BMI: body mass index; P/LP: pathogenic/likely pathogenic.

**Figure 2:**
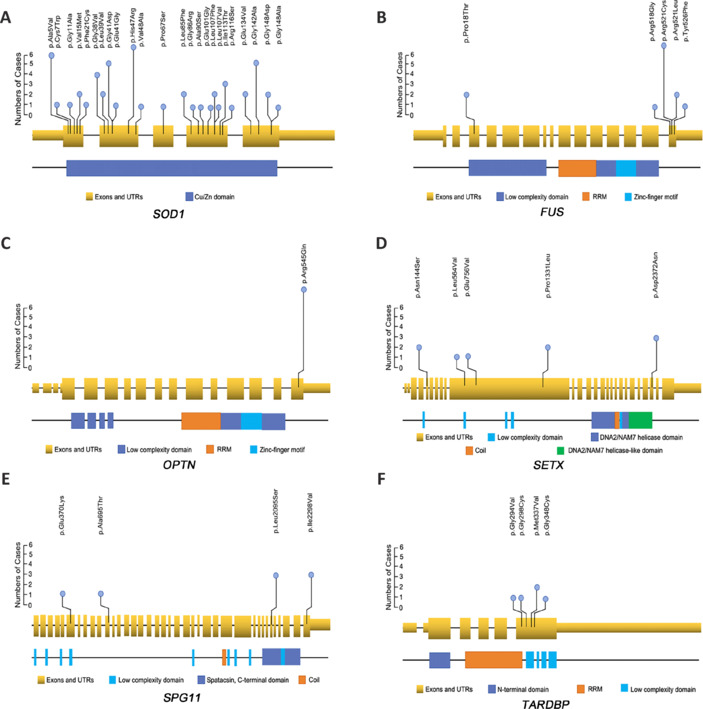
Lollipop plots showing the pathogenic and likely pathogenic variants identified in *SOD1* (A), *FUS* (B), *OPTN* (C), *SETX* (D), *SPG11* (E), and *TARDBP* (F) in our fALS cohort. The lollipop plots exhibit the mutational patterns of each gene in the cohort. Yellow bars indicate exons and untranslated regions, which are linked by gray lines representing introns. Colored bars depict protein domains and motifs. Each lollipop signifies a pathogenic or likely pathogenic mutation found in patients. The height of the lollipop corresponds to the total number of patients harboring the variant. ALS: Amyotrophic lateral sclerosis; fALS: familial amyotrophic lateral sclerosis; P/LP: pathogenic/likely pathogenic.

Overall, the six most frequently mutated genes in our Chinese cohort of 146 fALS patients were *SOD1* (28.6%), *FUS* (7.1%), *OPTN* (6.5%), *SETX* (5.4%), *SPG11* (4.2%), and *TARDBP* (2.4%) (**Additional Table 12**). The P/LP variants in these six genes are illustrated in **Figure 2**. SOD1 was identified as the most frequent pathogenic gene, accounting for 28.6% of our fALS cases (**Additional Table 12**). All other nonsynonymous variants were identified as VUSs. In the remaining 72 patients (36.9%), VUS mutations were detected by our screening approach. Notably, no pathogenic hexanucleotide repeat expansions in *C9ORF72* were detected in the full cohort of 244 fALS patients.

**Additional Table 12:** Genotype-phenotype correlation of 146 fALS index patients with P/LP variants in causative genes

Gene	No. of patients	Sex ratio	Age at onset, mean years (95% CI)	% spinal onset	% bulbar onset	Diagnostic delay, mean months (95% CI)	BMI, mean (95% CI)	Percent	Percent compared with other fALS populations	Percent in previous Chinese fALS	Ref
***SOD1***	48	1.4	43.5 (40.7-46.4)	100	0	28.6 (15.9-41.4)	22.6 (21.6-23.5)	28.6%	Korea 54.7%, Finland 42.9%, Sweden 35.6%, Japan 30.3-35.5%, Iran 35.3%, UK 24.4%, France 11.6-12.8%, Germany 11%, India 9.1%	25-42.9%	1-5
***FUS***	12	1.0	41.4 (33.6-49.2)	91.7	8.3	18.8 (0.709-36.8)	22.6 (20.4-24.7)	7.1%	Korea 11.1%, Japan 10.3%, Italy 1.9-7.5%, Spain 6.7%, Germany 2.4-4.3%, USA 1.4-4.1%	4.9-10%	1,4,6,7
***OPTN***	11	2.7	43.0 (36.1-49.9)	72.7	27.3	23.6 (7.55-39.7)	23.4 (20.7-26.1)	6.5%	Japan 3.8%, Germany 0.8%, Australia 0.5%, few in Caucasian population	0-2.3%	3,8-12
***SETX***	9	1.3	47.7 (39.3-56.0)	100	0	34.1 (5.85-62.4)	23.7 (21.1-26.4)	5.4%	Germany 2.4%, Japan 2%, France 0.9%	0-3.1%	2,3,9,13 15
***SPG11***	7	1.3	51.9 (37.9-65.8)	100	0	13.6 (3.98-23.2)	22.2 (19.5-24.9)	4.2%	/	/	/
***TARDBP***	4	4.0	53.4 (45.9-60.9)	80	20	18.4 (-2.13-38.9)	20.2 (17.9-22.5)	2.4%	Italy 0-12.5%, USA 0-3.9%, Germany 0-2.6%, Australia 1.9%	0-23.3%	8
***ALS2***	4	4.0	36.5(22.2-50.8)	100	0	31.5 (-48.7-112)	22.4 (16.8-28.0)	2.4%	Japan 2.0%, Germany 0.3%, Ireland 0/50	0-2.3%	9,13
***CCNF***	4	3.0	47.3 (28.5-66.0)	75	25	15.8 (-0.958-32.5)	23.6 (21.2-26.0)	2.4%	Australia 0.5%	0-2.56%	8
***SIGMAR1***	3	2.0	54.3 (46.3-62.3)	100	0	23.3 (-19.1-65.8)	24.2 (17.4-31.1)	1.8%	Korea 0/258	0/64, 0/15, 0/24	16
***ANXA11***	2	2.0	56.5	100	0	18.5 (-140-177)	21.6	1.2%	US 1.7%, France 1.5%, Germany 1.3%	0-2.3%	3,9,17-19
***FIG4***	1	Male	41	100	0	4	26.1	0.6%	Germany 0.5%	/	3
***HNRNPA* *1***	1	Male	56.5	0	100	6	21.8	0.6%	Italy 0/113, Australia 0/109, Netherlands 0/135	/	20-22
***DCTN1***	1	Female	59	0	100	56	18	0.6%	France 0.4%, Germany 0/382	3.6%	2,3,23
***ERBB4***	1	Male	48	0	100	8	23.2	0.6%	/	0-6%	24
***CHMP2B***	1	Male	46	0	100	13	22.9	0.6%	/	/	/
***NEK1***	1	Male	45	100	0	12	18.3	0.6%	European 3%	2.3%	14,15,25
**VUS**	37	1.3	51.8 (47.3-56.4)	81.1	18.9	36.6 (18.9-54.4)	23.8 (22.7-25.0)	22.0%	/	/	/
**Unknown**	21	3.2	49.5 (43.4-55.7)	66.7	33.3	33.0 (12.1-53.9)	22.6 (21.4-23.9)	12.5%	/	/	/

ALS: Amyotrophic lateral sclerosis; CI: confidence interval; P/LP: pathogenic/likely pathogenic; VUS: variant of uncertain significance.

### Genotype–phenotype correlation of patients with pathogenic/likely pathogenic variants in amyotrophic lateral sclerosis-causative genes

**Figure 3:**
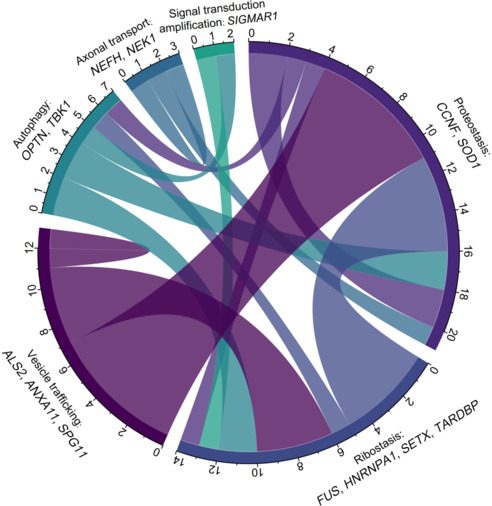
Interactions between biological processes implicated in ALS-causative genes of fALS. Chord diagram summarizing the interactions among biological processes (shown around the circle) implicated in ALS-causative genes of 26 fALS patients who carried two or more P/LP variants. Links between circles represent interactions. The scale on the circle indicates the number of patients who carry the gene participating in the indicated biological process. ALS: Amyotrophic lateral sclerosis; fALS: familial amyotrophic lateral sclerosis; P/LP: pathogenic/likely pathogenic.

We also explored the link between genotypes and phenotypes of ALS in our Chinese cohort, with detailed correlations presented in **Additional Table 12**. Mutations in all genes except *FUS* occurred more often in male than in female patients, especially in *TARDBP* and *ALS2* (male:female ratio, 4.0) (**Additional Table 12**). *ALS2* mutations, known as a main genetic factor in juvenile motor neuron diseases, presented with the earliest age of onset (mean, 36.5 years). The mean age of onset of fALS index patients who had no known P/LP variants was 49.5 years, while that of patients who carried VUSs was 51.8 years. Notably, the mean diagnostic delay from symptom onset in patients with no known mutated P/LP gene was 33 months, while that in patients carrying VUSs was 36.6 months (**Additional Table 13**). Interestingly, 17.8% of patients possessed two or more P/LP variants in ALS-causative genes. Details of the variants and clinical information are presented in **Additional Table 13**. Analyses of interactions between biological processes implicated in fALS-causative genes at the protein or gene level were illustrated using a chord diagram (**Figure 3**). This revealed that patients harboring variants in genes affecting proteostasis were more likely to also carry variants in genes that participate in vesicle tracking, illustrating the precise disease mechanism at the gene level.

### Burden analysis of the familial amyotrophic lateral sclerosis cohort

**Figure 4:**
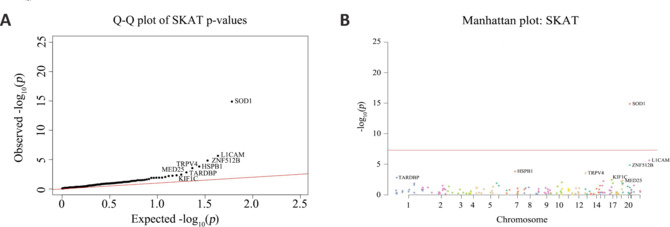
Gene burden analysis of fALS patients and in-house controls using the SKAT method. (A) Quantile-Quantile (Q‒Q) plot of single-nucleotide polymorphisms (SNPs) in the gene burden analysis (λ = 2.117). (B) Manhattan plot of SNPs in the gene burden analysis. fALS: Familial amyotrophic lateral sclerosis; Q‒Q plot: quantile‒quantile plot; SNP: single-nucleotide polymorphism.

Burden analysis of our fALS cohort was performed to ascertain whether there was a higher prevalence of uncommon mutations in any single gene (ALS-associated variant) or a decrease in such mutations among ALS patients (ALS-protective variant). We examined the distributions of infrequent mutations within the fALS group of 139 patients and the group of 1812 in-house controls using the SKAT method (Chen et al., 2021). Employing an MAF threshold of 0.01%, SKAT analysis showed that the rare variant burden of *SOD1* (*P* = 1.3e−15) was associated with a statistically significant risk of ALS disease. The *L1CAM* (*P* = 2.3e−6) and *ZNF512B* (*P* = 1.5e−5) genes were the second and third most enriched in rare variants, respectively, but the prevalences of such mutations did not reach statistical significance (**Figure 4** and **Additional Table 14**).

**Additional Table 14:** Burden analysis of rare variants (< 0.01%) in ALS-associated genes among 139 index patients with fALS and 1812 in-house controls

Gene	*P*-value
*SODI*	1.3e-15
*L1CAM*	2.3e-06
*ZNF512B*	1.5e-05
*HSPB1*	1.5e-04
*TRPV4*	2.8e-04
*TARDBP*	1.5e-03
*KIF1C*	3.7e-03
*MED25*	4.8e-03
*CRYM*	5.6e-03
*PRX*	6.3e-03

ALS: Amyotrophic lateral sclerosis; fALS: familial ALS.

## Discussion

In this study, we analyzed the clinical features and genetic spectrum of fALS in the Chinese mainland, based on a 15-year, clinic-based cohort of patients from 28 provinces. Several genome-wide NGS investigations of the mutation patterns of ALS-associated genes in fALS have been conducted in China, a country that accounts for a fifth of the global population. However, because these studies included limited numbers of fALS patients (Liu et al., 2019, 2021; Chen et al., 2020, 2021) and lacked long-term follow-up, we have yet to gain a comprehensive understanding of the genetic diversity and long-term correlation between genotype and phenotype in ALS. Our study findings represent the first characterization of the occurrence and development of fALS within familial clusters using a GBTM for ALSFRS-R score progression and, combined with data comparing fALS with sALS, may contribute to a deeper understanding of genotype–phenotype relationships in ALS.

In Chinese mainland, the mean age of onset in fALS patients (46.9 years) is 2.9 years younger than that in sALS patients (49.8 years) (Chen et al., 2015), in contrast to a 5.3-year difference between fALS and sALS patients in the United Kingdom (fALS, 56.2 years; sALS, 61.5 years) (Veldink, 2019). This discrepancy is thought to reflect the different genetic backgrounds within these populations. The diagnostic delay is 18.9 months earlier in fALS patients (30.9 months) than in sALS patients (49.8 months), while the proportions of patients with spinal onset and bulbar onset are similar in fALS and sALS (spinal-onset, fALS 85.4% *vs*. sALS 75.1%; bulbar-onset, fALS 14.6% *vs.* sALS 14.0%) (Chen et al., 2015). These findings demonstrated that fALS patients experience a distinctly earlier age of onset and slower progression than sALS patients.

**Figure 5:**
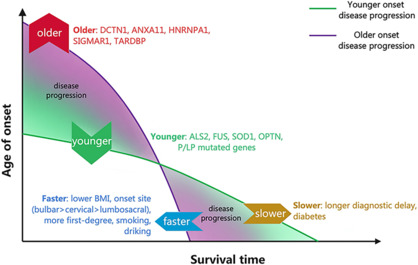
Hypothesis for the dynamic progression of disease based on ALSFRS-R scores in patients with fALS. Schematic illustration summarizing the dynamic changes in disease severity and functional scores that occur with disease progression. The fALS patients with mutations in *DCTN1*, *ANXA11*, *HNRNPA1*, *SIGMAR1*, and *TARDBP* tended to have an older age of onset, whereas those with mutations in P/LP genes, especially *ALS2*, *FUS*, *SOD1*, and *OPTN*, had a younger age of onset. The fALS patients with an earlier onset, longer diagnostic delay, higher BMI, fewer first-degree relatives, and spinal onset exhibited a slow progression of disease. By contrast, patients who had a later onset, shorter diagnostic delay, lower BMI, more first-degree relatives, bulbar onset, and a history of smoking and drinking exhibited rapid progression. ALSFRS-R: ALS functional rating scale-revised; BMI: body mass index; fALS: familial amyotrophic lateral sclerosis; P/LP: pathogenic/likely pathogenic.

Our hypothesis for the dynamic change in ALSFRS-R scores in the fALS group with rapid disease progression involves older age at onset, shorter diagnostic delay, lower BMI, bulbar onset, and one or more affected first-degree relatives (**Figure 5**). Interestingly, this group of patients also exhibited the highest proportion (64.52%) of riluzole users. In prognostic studies, the effect of riluzole on the survival of ALS patients has been a subject of controversy. While most studies suggested that riluzole is associated with extended survival, our previous sALS study did not observe this trend (Chen et al., 2015). Certain researchers have proposed that riluzole may merely decelerate, rather than halt, the degeneration of motor neurons, rendering it less effective for patients in advanced stages (Chen et al., 2016). Considering that the age at onset and disease severity are more varied in real-world cohort studies compared with randomized trials, our observation that the prognosis of the riluzole group was not better than that of the control group may be attributable to the inclusion of older and more severely affected patients in our cohort. The findings in our fALS cohort are consistent with those in sALS studies, indicating that older age at onset, shorter diagnostic delay, and lower BMI are associated with a trend towards faster progression (Chen et al., 2015). The comparison of clinical trajectories in fALS and sALS patients indicated that certain homogenous variables, including age of onset and time from onset to diagnosis, might differentially influence the disease progressions of fALS and sALS. The heterogeneous variables BMI, bulbar onset, and tumor history also may influence the progression of sALS and fALS; however, more validation is needed.

Our findings represent the only current resource for determining the influence of a detailed family history on ALS risk. Interestingly, we found that having one or more affected first-degree relatives was associated with faster progression. Another important finding that emerged from the data using the GBTM was that the number of P/LP gene variants did not influence the prognosis of fALS. Additionally, according to the classification of patients by possible, probable, or definitive fALS, the number of P/LP gene variants did not influence the age of onset, diagnostic delay, BMI, or fALS distribution, which is consistent with the results from the GBTM. These findings showed that having one or more affected first-degree relatives is more harmful than the genetic burden. One possible explanation for these results might be a relatively low penetrance within the overall population, but the rate of penetrance is significantly higher in symptomatic probands with affected first-degree relatives (Farrell et al., 2022). However, penetrance is gene and variant specific; for example, the penetrance of the *SOD1* A4V variant is **~**90% (Tang et al., 2018), but its incidence was rare in our Chinese cohort. These observations suggested that high penetrance of a genetic mutation, but not the number of mutations, contributes to the clinical development of fALS. Evidence from a pedigree study has also shown that, even when genetic mutations associated with ALS are absent in the proband, there remains an increased likelihood for first-degree relatives of ALS patients to develop ALS compared with the general population (Ryan et al., 2019). Furthermore, the highest estimated rates of heritability are consistently observed within mother-daughter pairs, indicating a previously unnoticed association between disease heritability and sex (Ryan et al., 2019). First-degree relatives of individuals with ALS may face unique psychosocial effects and challenges that can significantly impact their mental and emotional well-being. The knowledge of having a close family member with ALS often leads to heightened anxiety, depression, and fear of developing the disease themselves, particularly in fALS cases. This study underscored the importance of addressing psychosocial factors in the management and support of fALS-affected families.

In this comprehensive analysis of the genetic architecture in Chinese individuals with fALS, we focused on genes that are recognized as either pathogenic or likely pathogenic pending confirmation. We observed P/LP variants in 42 causative ALS genes in 60% (88 patients) of the 146 index patients, whereas 14% (21 patients) and 25% (37 patients) of the index patients remained genetically unexplained or carried VUSs, respectively. Notably, a German fALS cohort study on 301 families indicated that P/LP variants accounted for 49% of cases, while gene mutation negativity accounted for 43%, consistent with our cohort. Because only a fraction of cases can be attributed to Mendelian inheritance, a previously overlooked oligogenic model of ALS has surfaced (Shepheard et al., 2021). This model includes a small number of risk genes. In our study, 17.8% of 224 patients possessed two or more P/LP variants. A study conducted in Australia discovered that 7% of 616 sALS patients possessed two P/LP variants (McCann et al., 2020), whereas an Irish cohort study encompassing both fALS and sALS patients reported that only 2% of patients carried two or more P/LP variants for ALS (Ryan et al., 2018). Such results indicate that oligogenic ALS is more impactful in Chinese patients than in those of European descent (Kitzler et al., 2019). One possible explanation for these differences might be that *SOD1*, which ranks first in the mutation spectrum, is more likely to carry multiple mutations than other pathogenic genes. Our analysis revealed that the most prevalent clinical phenotypes among the affected individuals included in our study were behavioral variant FTD, followed by semantic dementia and primary progressive aphasia. This distribution aligns with the existing construct of ALS-FTD phenotypes. On the basis of documented genetic alterations, relatives of ALS patients who exhibited genetic mutations in *C9ORF72*, followed by *MAPT* and *GRN*, were more likely to present with FTD (Greaves and Rohrer, 2019; Costa et al., 2020). However, variants of these genes were not detected in FTD patients in our cohort, possibly because no pathogenic hexanucleotide repeat expansions in *C9ORF72* were detected in our cohort.

Valuable insights into the genetic architecture of fALS are illustrated in **Additional Table 12**, which compares the percentages of P/LP variants in causative genes detected in our Chinese cohort with those in previously studied European and Chinese populations. Surprisingly, *OPTN* ranked third (6.5%) in the Chinese fALS mutation spectrum. Additionally, our data elucidated the connection between genotypic and phenotypic manifestations of ALS within the familial Chinese population. Overall, *SOD1*, *FUS*, *OPTN*, and *SETX* were the four highest ranked genes in our Chinese fALS cohort. By comparison, the four highest ranked genes in European fALS cohorts were *C9ORF72*, *SOD1*, *TARDBP*, and *FUS* (Zou et al., 2017; Muller et al., 2018; Corcia et al., 2021), emphasizing the significance of assessing the genetic spectrum of disease in different ethnicities. According to our genetic analysis, the most common pathogenic gene in Chinese fALS patients was *SOD1*, consistent with previous studies. *SOD1* exhibits a high proportion of the disease-causing mutations, a wide variety of mutation sites, and a diverse clinical phenotype among fALS patients, and is also the first therapeutic target of ALS-targeted gene therapy to be approved by the U.S. Food and Drug Administration (Miller et al., 2022). Mutations in *L1CAM* can lead to changes in the structure or function of L1 cell adhesion molecules, which can affect the normal development of the nervous system (Altevogt et al., 2016). Although there is no reported evidence of a direct correlation between *L1CAM* and ALS in the literature, interestingly, the decreased expression of LICAM protein observed in the cerebrospinal fluid of ALS patients (Oh et al., 2023) may be a secondary phenomenon of neuronal cell death. The protein expressed by *ZNF512B*, which regulates cell growth and differentiation, belongs to the zinc finger protein family and contains multiple C2H2-type zinc finger domains. It has been suggested that decreased *ZNF512B* expression increases susceptibility to ALS (Iida et al., 2011). Subsequent studies on the association of clinical phenotypes with this gene in Japan and China found that ALS patients with *ZNF512B* mutations had shorter survival (Tetsuka et al., 2013; Jiang et al., 2021). Data from our cohort support the link between *ZNF512B* mutation and ALS susceptibility in the Chinese population. However, this link was not observed in European or American populations, and the potential prognostic effect of ZNF512B mutations in ALS patients was not confirmed (Chio et al., 2023). Therefore, although *L1CAM* and *ZNF512B* may have potential as novel risk factors for ALS, additional cohort studies and biological validation are needed. Synthesizing insights from the most common ALS-associated genes (*SOD1*, *C9ORF72*, *TARDBP*, *FUS*), we propose distinct hypothetical mechanisms that could account for a more rapid progression of damage in ALS. Mutations in *SOD1* enhance the production of reactive oxygen species, leading to oxidative stress and motor neuron apoptosis (Chia et al., 2018). The hexanucleotide repeat expansions in *C9ORF72* form nuclear foci that interfere with RNA processing and transport, thereby disrupting gene expression and protein synthesis (Balendra and Isaacs, 2018). Additionally, aberrant TDP-43 and FUS proteins with loss of standard functions cause dysregulation in RNA processing and transport, directly impairing neuronal function and survival (Taylor et al., 2016). Future research using cellular and animal models could be crucial in validating these hypotheses and exploring potential therapeutic interventions.

This study has some limitations. First, because of continuous advancements in sequencing technology during the 15-year duration of this research, different methods were employed to sequence DNA samples from different participants; namely, Sanger sequencing and genome-wide NGS for patients and in-house controls. This resulted in a lower rate of detection of pathogenic gene variants in the early phase of the study, thereby leading to a lower overall rate of detection in our results. Second, all of the data were collected from a single center; thus, the regional distribution of fALS may have been influenced by the patients’ economic levels and travel distances from the center. Third, we did not have follow-up tracking for relatives who were found to carry pathogenic genes but had not yet developed the disease. One future aim is to identify the characteristics of those who remain asymptomatic over time, and the other is to discover any pathophysiological changes that occur prior to the appearance of symptoms in patients with a family history of ALS.

In conclusion, we conclusively confirmed the clinical features and genetic spectrum of fALS over a 15-year follow-up of a clinical cohort of patients in the Chinese mainland, from both epidemiological and genetic perspectives. Our research findings deepen the understanding of fALS disease progression, offering valuable insights for future clinical trials. Importantly, the study also conclusively identified both shared (age at onset and time from onset to diagnosis) and distinct (BMI, bulbar onset, and tumor history) variables that impact disease progression in both fALS and sALS. The data patterns identified here can be used to evaluate the clinical prognosis of ALS, and may support the development of genotype-specific treatment approaches.

## Additional files:

***Additional Figure 1:*** *Study flow diagram.*

Additional Figure 1 Study flow diagram.
ACMG: American College of Medical Genetics and Genomics; ALS: amyotrophic lateral sclerosis; fALS: familial amyotrophic lateral sclerosis; PCR: polymerase chain reaction.

***Additional Figure 2:*** *Comparison of clinical trajectories generated using the original cohort and bootstrapped data from the four disease progression groups.*

Additional Figure 2 Comparison of clinical trajectories generated using the original cohort and bootstrapped data from the four disease progression groups.
The trajectories obtained from the internal validation cohort using the bootstrap procedure were consistent with the original results from our patient cohort: red (rapid group), blue (moderate group), green (slow group), and black (relatively stable group). Solid lines: patient cohort; dotted lines: internal validation cohort.

***Additional Table 1:*** *Performance of group-based trajectory models for ALSFRS-R according to the number of groups and trajectory shapes.*

Additional Table 1 Performance of group-based trajectory models for ALSFRS-R according to the number of groups and trajectory shapes

***Additional Table 2:*** *Basic characteristics of 42 ALS-causative genes investigated in this study.*

***Additional Table 3:*** *Geographical distribution of fALS patients in China.*

***Additional Table 4:*** *Demographic and clinical characteristics of fALS patients.*

***Additional Table 5:*** *Results of the trajectory analysis of the bootstrap samples versus the original results.*

***Additional Table 6:*** *Impact of urban and rural areas on the onset of fALS.*

***Additional Table 7:*** *Comparison of the trajectory groups of patients with fALS and sALS.*

***Additional Table 8:*** *Baseline characteristics of sALS patients in the different ALSFRS trajectory groups.*

***Additional Table 9:*** *Associations between respiratory insufficiency or death and the trajectory groups according to the multivariable Cox proportional hazards model.*

***Additional Table 10:*** *Survival of fALS patients with different gene mutations.*

***Additional Table 11:*** *Genotype‒phenotype correlations in 244 index patients with P/LP variants in major ALS-causative genes.*

***Additional Table 12:*** *Genotype‒phenotype correlation of 146 fALS index patients with P/LP variants in causative genes.*

***Additional Table 13:*** *Information on the 26 ALS patients who carried two or more P/LP variants in ALS-causative genes.*

Additional Table 13 Information on the 26 ALS patients who carried two or more P/LP variants in ALS-causative genes

***Additional Table 14:*** *Burden analysis of rare variants (< 0.01%) in ALS-associated genes among 139 index patients with fALS and 1812 in-house controls.*

## Data Availability

*All relevant data are within the paper and its Additional files*.
