# Supplementary material for: Comprehensive clinical and genetic architecture of familial amyotrophic lateral sclerosis in China: A 15-year cohort study with 302 families
Source: Neural Regen Res. 2025 Jan 13;21(6):2573–9. doi: 10.4103/NRR.NRR-D-24-00701 (PMC13211834; doi:10.4103/NRR.NRR-D-24-00701)
Supplement: Supplementary file 1 [file NRR-21-2573_Suppl1.pdf]

Additional Tables

Additional Table 1 Performance of group-based trajectory models for ALSFRS-R according to the number of groups and trajectory shapes

Highest order of each group	BIC[a]	Proportion of the smallest group (%)	*P*[b]
Three groups			
1,1,1	-4209.19	27.04973	< 0.0001, < 0.0001, < 0.0001
1,1,2	-4182.26	25.43474	< 0.0001, < 0.0001, < 0.0001
1,1,3	-4109.36	16.03049	< 0.0001, < 0.0001, < 0.0001
1,2,1	-4183.14	22.92373	< 0.0001, < 0.0001, < 0.0001
1,2,2	-4172.35	13.16626	< 0.0001, < 0.0001, < 0.0001
1,2,3	-4160.81	11.90202	< 0.0001, < 0.0001, < 0.0001
1,3,1	-4181.02	11.56699	< 0.0001, < 0.0001, < 0.0001
1,3,2	-4156.35	12.31747	< 0.0001, < 0.0001, < 0.0001
1,3,3	-4158.10	11.92621	< 0.0001, < 0.0001, < 0.0001
2,1,1	-4180.39	22.97854	< 0.0001, < 0.0001, < 0.0001
2,1,2	-4141.34	12.71946	< 0.0001, < 0.0001, < 0.0001
2,1,3	-4139.62	23.80996	< 0.0001, < 0.0001, < 0.0001
2,2,1	-4148.58	23.76698	< 0.0001, < 0.0001, < 0.0001
2,2,2	-4119.54	12.68621	< 0.0001, < 0.0001, < 0.0001
2,2,3	-4106.10	14.32560	< 0.0001, < 0.0001, < 0.0001
2,3,1	-4133.51	12.28025	< 0.0001, < 0.0001, < 0.0001
2,3,2	-4169.48	13.71347	< 0.0001, 0.5619, < 0.0001
2,3,3	-4105.05	12.85374	< 0.0001, < 0.0001, 0.0033
3,1,1	-4167.50	24.94394	< 0.0001, < 0.0001, < 0.0001
3,1,2	-4144.68	12.76833	0.0001, < 0.0001, < 0.0001
3,1,3	-4110.48	14.01002	< 0.0001, < 0.0001, < 0.0001
3,2,1	-4149.94	13.48749	< 0.0001, < 0.0001, < 0.0001
3,2,2	-4103.69	14.52807	< 0.0001, < 0.0001, < 0.0001
3,2,3	-4118.12	14.72060	< 0.0001, < 0.0001, < 0.0001
3,3,1	-4124.07	13.79238	< 0.0001, < 0.0001, < 0.0001
3,3,2	-4105.02	13.10300	< 0.0001, < 0.0001, < 0.0001
3,3,3	-4064.18	14.50605	< 0.0001, < 0.0001, 0.0016
Four groups			
1,1,1,1	-4100.83	14.84376	< 0.0001, < 0.0001, < 0.0001, < 0.0001

1,1,1,2	-4092.60	13.17725	< 0.0001, < 0.0001, < 0.0001, < 0.0001
1,1,1,3	-4093.63	14.13314	< 0.0001, < 0.0001, < 0.0001, < 0.0001
1,1,2,1	-4092.60	13.17726	< 0.0001, < 0.0001, < 0.0001, < 0.0001
1,1,2,2	-4087.43	13.67972	< 0.0001, < 0.0001, < 0.0001, 0.0003
1,1,2,3	-4089.79	13.43563	< 0.0001, < 0.0001, 0.0004, 0.3274
1,1,3,1	-4099.65	14.92666	< 0.0001, < 0.0001, 0.7040, < 0.0001
1,1,3,2	-4108.04	8.12341	< 0.0001, < 0.0001, < 0.0001, < 0.0001
1,1,3,3	-4107.85	8.23071	< 0.0001, < 0.0001, < 0.0001, 0.0144
1,2,1,1	-4114.59	8.34191	< 0.0001, < 0.0001, < 0.0001, < 0.0001
1,2,1,2	-4030.83	13.13309	< 0.0001, < 0.0001, < 0.0001, < 0.0001
1,2,1,3	-4082.86	1.26817	< 0.0001, < 0.0001, < 0.0001, < 0.0001
1,2,2,1	-4089.16	13.59964	< 0.0001, < 0.0001, 0.0078, < 0.0001
1,2,2,2	-4099.60	5.95653	< 0.0001, < 0.0001, < 0.0001, < 0.0001
1,2,2,3	-4049.87	14.71255	< 0.0001, < 0.0001, < 0.0001, < 0.0001
1,2,3,1	-4089.02	13.60080	< 0.0001, < 0.0001, 0.1614, < 0.0001
1,2,3,2	-4133.14	2.89542	< 0.0001, < 0.0001, < 0.0001, < 0.0001
1,2,3,3	-4094.56	1.85807	< 0.0001, < 0.0001, < 0.0001, < 0.0001
1,3,1,1	-4097.16	13.49077	< 0.0001, 0.6452, < 0.0001, < 0.0001
1,3,1,2	-4017.74	14.95545	< 0.0001, < 0.0001, < 0.0001, < 0.0001
1,3,1,3	-4014.80	12.74633	< 0.0001, < 0.0001, < 0.0001, < 0.0001
1,3,2,1	-4113.75	3.44792	< 0.0001, 0.9467, < 0.0001, < 0.0001
1,3,2,2	-4064.89	12.56244	< 0.0001, < 0.0001, < 0.0001, < 0.0001
1,3,2,3	-4015.17	14.83016	< 0.0001, < 0.0001, < 0.0001, 0.4435
1,3,3,1	-4088.44	14.18770	< 0.0001, < 0.0001, 0.0132, < 0.0001
1,3,3,2	-4013.20	12.79948	< 0.0001, < 0.0001, < 0.0001, < 0.0001
1,3,3,3	-4015.57	14.63035	< 0.0001, < 0.0001, < 0.0001, 0.0344
2,1,1,1	-4037.73	14.54085	< 0.0001, < 0.0001, < 0.0001, < 0.0001
2,1,1,2	-4030.83	13.13323	< 0.0001, < 0.0001, < 0.0001, < 0.0001
2,1,1,3	-4033.32	13.25881	< 0.0001, < 0.0001, < 0.0001, < 0.0001
2,1,2,1	-4030.83	13.13316	< 0.0001, < 0.0001, < 0.0001, < 0.0001
2,1,2,2	-4029.35	13.15152	< 0.0001, < 0.0001, < 0.0001, 0.0002
2,1,2,3	-4031.11	13.91216	< 0.0001, < 0.0001, 0.0002, 0.0120
2,1,3,1	-4037.65	14.44693	< 0.0001, < 0.0001, 0.7900, < 0.0001
2,1,3,2	-4028.14	14.45219	< 0.0001, < 0.0001, 0.4445, < 0.0001

2,1,3,3	-4083.75	14.25866	< 0.0001, < 0.0001, 0.0203, 0.8313
2,2,1,1	-4074.57	13.60674	< 0.0001, < 0.0001, < 0.0001, < 0.0001
2,2,1,2	-4128.72	2.05418	0.0434, < 0.0001, < 0.0001, < 0.0001
2,2,1,3	-4147.59	0.00000	1.0000, < 0.0001, < 0.0001, 0.0001
2,2,2,1	-4021.25	13.24557	< 0.0001, < 0.0001, < 0.0001, < 0.0001
2,2,2,2	-4108.02	4.92476	< 0.0001, < 0.0001, < 0.0001, < 0.0001
2,2,2,3	-4024.84	13.22701	<0.0001, < 0.0001, < 0.0001, 0.5469
2,2,3,1	-4083.98	11.10239	0.0104, < 0.0001, < 0.0001, 0.5469
2,2,3,2	-4061.45	10.20978	< 0.0001, < 0.0001, < 0.0001, < 0.0001
2,2,3,3	-4066.40	9.14614	< 0.0001, < 0.0001, < 0.0001, < 0.0001
2,3,1,1	-4030.20	13.10025	< 0.0001, < 0.0001, < 0.0001, < 0.0001
2,3,1,2	-4013.94	12.88926	< 0.0001, < 0.0001, < 0.0001, < 0.0001
2,3,1,3	-4103.34	1.77425	0.0530, 0.0001, < 0.0001, < 0.0001
2,3,2,1	-4016.42	14.58091	0.0001, < 0.0001, < 0.0001, < 0.0001
2,3,2,2	-4085.32	11.71211	< 0.0001, 0.0003, < 0.0001, < 0.0001
2,3,2,3	-4095.66	0.00006	0.9993, < 0.0001, < 0.0001, 0.0072
2,3,3,1	-4036.10	13.11916	< 0.0001, 0.2725, 0.7988, < 0.0001
2,3,3,2	-4114.59	0.00000	1.0000, 0.0003, < 0.0001, < 0.0001
2,3,3,3	-4075.35	0.98162	0.0196, < 0.0001, < 0.0001, < 0.0001
3,1,1,1	-4026.63	14.83398	< 0.0001, < 0.0001, < 0.0001, < 0.0001
3,1,1,2	-4155.54	4.04819	0.0270, < 0.0001, < 0.0001, < 0.0001
3,1,1,3	-4014.80	12.74633	< 0.0001, < 0.0001, < 0.0001, < 0.0001
3,1,2,1	-4020.93	14.92976	< 0.0001, < 0.0001, < 0.0001, < 0.0001
3,1,2,2	-4020.48	13.66461	< 0.0001, < 0.0001, < 0.0001, 0.0001
3,1,2,3	-4016.70	14.75012	0.0130, < 0.0001, < 0.0001, < 0.0001
3,1,3,1	-4040.88	13.58558	< 0.0001, < 0.0001, 0.8203, < 0.0001
3,1,3,2	-4010.18	14.40051	< 0.0001, < 0.0001, < 0.0001, 0.0002
3,1,3,3	-4015.24	13.89195	< 0.0001, < 0.0001, < 0.0001, 0.9660
3,2,1,1	-4022.63	12.93481	< 0.0001, < 0.0001, < 0.0001, < 0.0001
3,2,1,2	-4022.09	12.84046	< 0.0001, < 0.0001, < 0.0001, 0.0002
3,2,1,3	-4004.26	14.38072	< 0.0001, < 0.0001, < 0.0001, < 0.0001
3,2,2,1	-4013.94	12.88931	< 0.0001, < 0.0001, < 0.0001, < 0.0001
3,2,2,2	-4007.13	14.77184	< 0.0001, < 0.0001, < 0.0001, < 0.0001
3,2,2,3	-4064.12	3.03245	< 0.0001, < 0.0001, < 0.0001, < 0.0001

3,2,3,1	-4059.11	10.46345	< 0.0001, < 0.0001, < 0.0001, < 0.0001
3,2,3,2	-4099.74	1.25702	0.0044, < 0.0001, < 0.0001, < 0.0001
3,2,3,3	-4059.45	11.24182	< 0.0001, < 0.0001, < 0.0001, < 0.0001
3,3,1,1	-4028.29	12.78779	< 0.0001, 0.1412, < 0.0001, < 0.0001
3,3,1,2	-4108.48	6.17622	< 0.0001, < 0.0001, < 0.0001, < 0.0001
3,3,1,3	-4120.00	3.14777	< 0.0001, < 0.0001, < 0.0001, < 0.0001
3,3,2,1	-4032.56	14.03838	< 0.0001, < 0.0001, < 0.0001, < 0.0001
3,3,2,2	-4058.23	1.72428	< 0.0001, < 0.0001, < 0.0001, < 0.0001
3,3,2,3	-4055.48	0.86110	0.9683, < 0.0001, < 0.0001, < 0.0001
3,3,3,1	-4115.56	2.23702	0.0474, < 0.0001, < 0.0001, < 0.0001
3,3,3,2	-4052.48	1.39474	0.0005, < 0.0001, < 0.0001, < 0.0001
3,3,3,3	-4065.82	5.43213	< 0.0001, < 0.0001, < 0.0001, 0.0001

ALSFRS-R: revised ALS functional rating scale; ALS, amyotrophic lateral sclerosis. [a]BIC for the total number of participants. [b]P for the highest order of each subgroup
