# Supplementary figures and images for: Comprehensive clinical and genetic architecture of familial amyotrophic lateral sclerosis in China: A 15-year cohort study with 302 families
Source: Neural Regen Res. 2025 Jan 13;21(6):2573–9. doi: 10.4103/NRR.NRR-D-24-00701 (PMC13211834; doi:10.4103/NRR.NRR-D-24-00701)

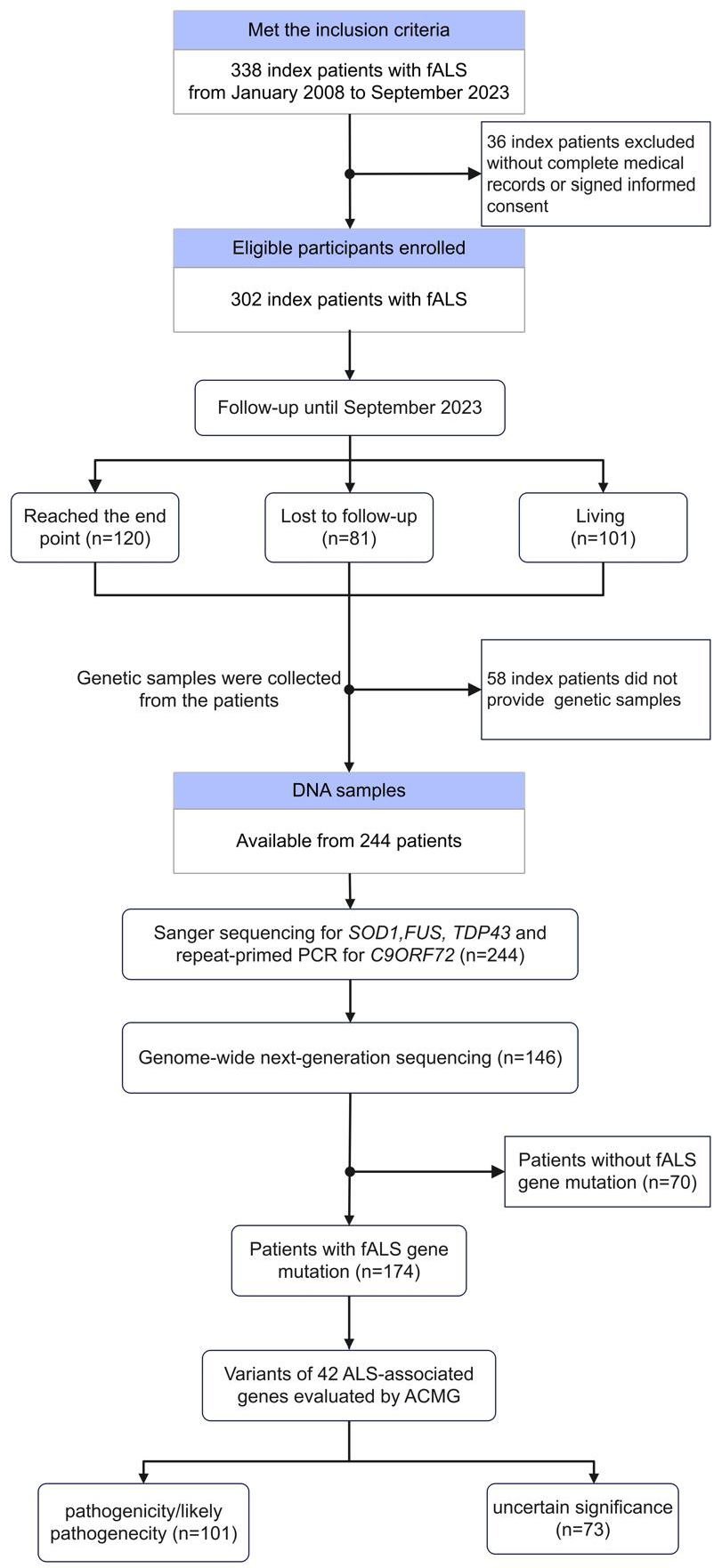

Supplement: Supplementary file 2 [file NRR-21-2573_Suppl1.tif]

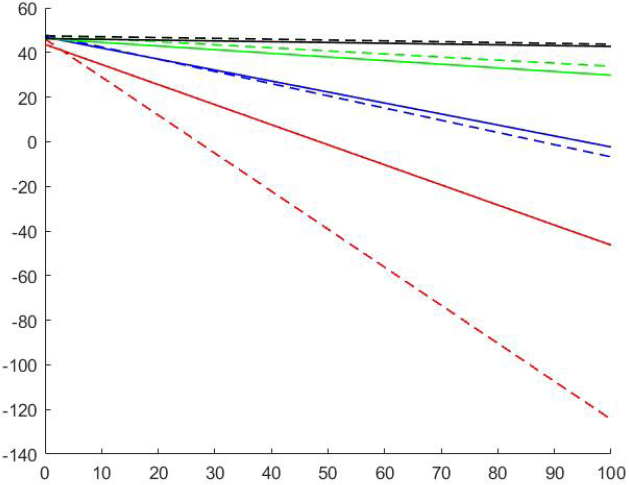

Supplement: Supplementary file 4 [file NRR-21-2573_Suppl2.tif]
