# Supplementary material for: Comprehensive clinical and genetic architecture of familial amyotrophic lateral sclerosis in China: A 15-year cohort study with 302 families
Source: Neural Regen Res. 2025 Jan 13;21(6):2573–9. doi: 10.4103/NRR.NRR-D-24-00701 (PMC13211834; doi:10.4103/NRR.NRR-D-24-00701)
Supplement: Supplementary file 3 [file NRR-21-2573_Suppl2.pdf]

Additional Table 13 Information on the 26 ALS patients who carried two or more P/LP variants in ALS-causative genes

No.	Genes	cDNA	Amino acid change	Variant type	Diagnostic delay from symptom onset (mon)	Survival status	Duration (mon)	Onset location
1	*NEFH*	c.1138G>A	p.Ala380Thr	Missense	9	Death	64	Upper limb
	NEK1	c.1618C>T	p.Arg540*	Stop-gain				
2	*SOD1*	c.319C>T	p.Leu107Phe	Missense	6	Death	20	Upper limb
	TBK1	c.314A>G	p.Tyr105Cys	Missense				
3	*SOD1*	c.268G>T	p.Ala90Ser	Missense	7	Alive	13	Upper limb
	SOD1	c.269C>T	p.Ala90Val	Missense				
4	*SOD1*	c.446T>C	p.Val149Ala	Missense	3	Loss	9	Upper limb
	ALS2	c.475G>A	p.Glu159Lys	Missense				
5	*SOD1*	c.341T>C	p.Ile114Thr	Missense	6	Loss	13	Upper limb
	ALS2	c.3517G>A	p.Glu1173Lys	Missense				
6	*SOD1*	c.43G>A	p.Val15Met	Missense	8	Loss	15	Lower limb
	SPG11	c.6892A>G	p.Ile2298Val	Missense				
7	*SOD1*	c.43G>A	p.Val15Met	Missense	35	Death	81	Lower limb
	SETX	c.431A>G	p.Asn144Ser	Missense				
	SPG11	c.6892A>G	p.Ile2298Val	Missense				
8	*OPTN*	c.1634G>A	p.Arg545Gln	Missense	6	Loss	24	Medulla oblongata
	HNRNPA1	c.847G>A	p.Gly283Arg	Missense				
9	*OPTN*	c.1634G>A	p.Arg545Gln	Missense	21	Death	73	Lower limb
	CCNF	c.1810T>A	p.Phe604Ile	Missense				
10	*SETX*	c.7114G>A	p.Asp2372Asn	Missense	10	Loss	13	Upper limb
	SPG11	c.6284T>C	p.Leu2095Ser	Missense				
	ALS2	c.3517G>A	p.Glu1173Lys	Missense				

11	*FUS*	c.1561C>T	p.Arg521Cys	Missense	3	Alive	9	Medulla oblongata
	CCNF	c.1810T>A	p.Phe604Ile	Missense				
12	*TARDBP*	c.1009A>G	p.Met337Val	Missense	12	Death	87	Upper limb
	SIGMAR1	c.622C>T	p.Arg208Trp	Missense				
13	*SOD1*	c.32G>C	p.Gly11Ala	Missense	12	Death	16	Lower limb
	SETX	c.1690T>G	p.Leu564Val	Missense				
14	*SOD1*	c.140A>G	p.His47Arg	Missense	40	Alive	114	Lower limb
	FUS	c.52C>A	p.Pro18Thr	Missense				
15	*SOD1*	c.140A>G	p.His47Arg	Missense	102	Alive	176	Lower limb
	FUS	c.52C>A	p.Pro18Thr	Missense				
16	*SOD1*	c.125G>A	p.Gly42Asp	Missense	115	Death	126	Upper limb
	SETX	c.7114G>A	p.Asp2372Asn	Missense				
17	*FUS*	c.1577A>T	p.Tyr526Phe	Missense	6	Death	50	Upper limb
	SPG11	c.6892A>G	p.Ile2298Val	Missense				
18	*FUS*	c.1552A>G	p.Arg518Gly	Missense	6	Death	35	Lower limb
	OPTN	c.1634G>A	p.Arg545Gln	Missense				
19	*SOD1*	c.140A>G	p.His47Arg	Missense	27	Alive	91	Lower limb
	CCNF	c.1810T>A	p.Phe604Ile	Missense				
20	*SOD1*	c.112G>A	p.Gly38Arg	Missense	64	Alive	127	Upper limb
	OPTN	c.1634G>A	p.Arg545Gln	Missense				
21	*SOD1*	c.131A>G	p.His44Arg	Missense	11	Alive	25	Lower limb
	NEK1	c.859C>G	p.Pro287Ala	Missense				
22	*SOD1*	c.115C>G	p.Leu39Val	Missense	14	Loss	17	Lower limb
	SPG11	c.6284T>C	p.Leu2095Ser	Missense				

23	*FUS*	c.1562G>T	p.Arg521Leu	Missense	8	Alive	11	Upper limb
	OPTN	c.1634G>A	p.Arg545Gln	Missense				
24	*SOD1*	c.140A>G	p.His47Arg	Missense	107	Alive	112	Lower limb
	ALS2	c.3517G>A	p.Glu1173Lys	Missense				
25	*SOD1*	c.115C>G	p.Leu39Val	Missense	6	Alive	9	Upper limb
	ANXA11	c.962C>A	p.Thr321Asn	Missense				
26	*SIGMAR1*	c.622C>T	p.Arg208Trp	Missense	43	Alive	77	Lower limb
	OPTN	c.1634G>A	p.Arg545Gln	Missense				

ALS: amyotrophic lateral sclerosis; P/LP: pathogenic/likely pathogenic.
